# Impact of flow regime on the performance of anti-biofouling coatings

**DOI:** 10.1038/s41598-023-36736-7

**Published:** 2023-06-12

**Authors:** Venkatesh Pulletikurthi, Helber Antonio Esquivel-Puentes, Shyuan Cheng, Leonardo P. Chamorro, Luciano Castillo

**Affiliations:** 1 School of Mechanical Engineering, Purdue University, West Lafayette, IN 47906 USA; 2 Department of Agricultural and Biological Engineering, Purdue University, West Lafayette, IN 47906 USA; 3 Department of Mechanical Science and Engineering, University of Illinois Urbana-Champaign, IL 61801 Champaign, USA

**Keywords:** Engineering, Mechanical engineering

## Abstract

Biofouling poses significant challenges for marine transportation due to increased skin drag, which results in increased fuel cost and associated $\text {CO}_2$ emissions. Current antifouling methods involving polymer coating, biocides, and self-depleting layers harm marine ecosystems and contribute to marine pollution. Significant advancements have resulted in using bioinspired coatings to address this issue. However, prior investigations have predominantly focused on wettability and adhesion aspects, resulting in a limited understanding of the impact of flow regime on bioinspired structure patterns for antifouling. We conducted comprehensive experiments with two bioinspired coatings^1^ under laminar and turbulent flow regimes and compared them with a smooth surface. The two coatings are composed of regular arrangements of micropillars measuring 85 μm in height and spaced at 180 μm (pattern A) and 50 μm high micropillars spaced at 220 μm (pattern B). Theoretical arguments indicate that wall-normal velocity fluctuations near the micropillars’ top significantly contribute to reducing the onset of biofouling under turbulence compared to the smooth surface. Pattern A coating can effectively decrease biofouling by 90% for fouling sizes exceeding 80 microns when compared to a smooth surface subjected to a turbulent flow regime. The coatings exhibited comparable anti-biofouling properties under a laminar flow. Also, the smooth surface experienced substantially higher biofouling under laminar flow compared to turbulent conditions. This underscores how the effectiveness of anti-biofouling approaches is critically influenced by the flow regime.

## Introduction

The accumulation of organisms on submerged surfaces, or biofouling, has been shown to have a significant negative impact on pollution by contributing to greenhouse gas emissions, such as carbon dioxide (CO_2_). It has been estimated that the world’s marine fleet would have produced an additional 0.5 million tons of CO_2_ in 1986 if fouled, according to a study by Townsin et al.^2^. Recent research by the International Maritime Organization (IMO) in 2022^3^ indicates that even a relatively thin layer of biofouling, such as a 0.5 mm slime covering just half of a ship’s surface, can result in a significant increase of 25–30% in CO_2_ emissions. This highlights the need for effective measures to prevent biofouling and reduce its impact on pollution.

The economic impact of biofouling on naval fleets has been a critical subject over the years. Callow et al.^4^ conducted an extended economic analysis of the entire US Navy fleet based on the economic impact of biofouling on Arleigh Burke DDG-51 destroyers. Their findings suggest that biofouling generates an annual cost of $180–260 million (USDA). This underscores the importance of addressing biofouling to reduce not only environmental impacts but also economical costs. Biofouling poses a significant problem for various industries, particularly shipping and marine engineering, where it can lead to increased fuel consumption, machinery damage, and introducing invasive species to new environments^5^. Biofouling can be characterized into micro and macro fouling^4,6,7^, although they are indistinguishable in the fouling process, which involves a series of organic matter depositions following a predator-prey sequence^8^. Microorganisms attach themselves to surfaces using bio-adhesives that flow into surface imperfections and cure to create a secure mechanical lock^9^. Several techniques have been developed to combat biofouling, including paint based on tin-based polymers, controlled self-depleting layers, and hybrid-TBT-free paints. However, these methods have drawbacks, such as harming marine ecosystems or requiring regular recoating^10^.

To overcome various challenges in surface manipulation, scientists have turned to biomimicry and bioinspiration technologies, seeking inspiration from a wide range of organisms, from corals and plants to large mammals like sharks^11^. Among these, technologies inspired by the skin of various shark species, such as the spinner, Galapagos, and Mako, have been particularly popular due to their drag reduction properties^12,13^. Researchers have investigated the antifouling properties of bioinspired and biomimetic technologies that utilize riblet structures found on, e.g., shark skin^13^. These technologies incorporate diverse patterns and shapes, including the so-called sharklet coined by Hoipkemier-Wilson et al.^14^. This pattern features a placoid structure with dimensions of 4 μm in height and 2 μm in width, with a spacing of 2 μm, resulting in a significant reduction in settlement of Ulva spores. Further research has examined other patterns, such as 2 μm pillars, 2 μm ridges, and combinations of pillars and triangles^15–17^. Interestingly, all these patterns were found to reduce Ulva spore settlement compared to surfaces without any patterns. The antifouling effects observed in these studies can be attributed to surface wettability and prevention mechanisms, which involve releasing chemicals to prevent fouling. See also, Liu et al.^18^ for discussion on adhesion models at micro and nano scales.

Bioinspired surface modifications, such as mushroom-shaped microstructures, have demonstrated significant impacts on various areas, including heat transfer^19^, tribology^20^, and aerodynamics^1^. In particular, Petersen et al.^21^ implemented a hybrid antifouling approach by combining a mushroom-shaped surface topology with a silicone-based fouling release material, which has undergone field testing to prevent the attachment of barnacles. They showed that this hybrid approach effectively prevented the adhesion of macro fouling. However, to the best of the authors’ knowledge, the role of flow condition and their interaction with bioinspired structures remain open problem.

Previous research has demonstrated the efficacy of bioinspired structures derived from various animal and plant species, such as corals, porpoises, whales, sharks, crustose coraline algae, and red algae, in reducing biofouling^22^. The mechanism underlying this effect depends on the pattern and spacing of these microstructures. However, the success of various shapes, such as triangles, pillars, and ridges, in reducing biofouling is contingent on specific spacing and height, underscoring the crucial role of flow physics in addition to wetting and adhesion by bioorganisms.

**Figure 1:**
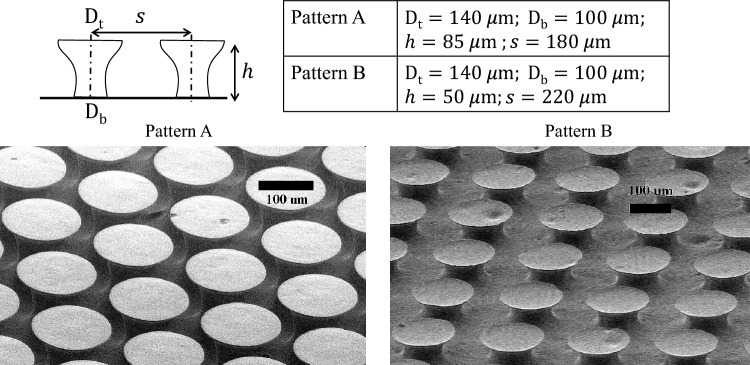
Micro-diverging pillars of base stalk diameter $D_b=100$ μm, and top diameter, $D_t= 140$ μm. with two different patterns: Pattern A with elements height of $h=85$ μm and center-to-center distance $s=180$ μm, and Pattern B with elements height of $h=50$ μm and center-to-center distance $s=220$ μm; the horizontal, black scale bar indicates 100 μm.

This article examines, for the first time, the unique impact of flow conditions on anti-biofouling and the corresponding mechanisms. The study employed commercially available bioinspired structures as micro-diverging pillars, as illustrated in Fig.1, utilizing two patterns of bioinspired structures. Both patterns feature a tip diameter of 140 μm and a base diameter of 100 μm. The disparity between these two patterns is their spacing and height, as indicated in Fig.1. Pattern A denotes the configuration with a height of 85 μm and a spacing of 180 μm, while pattern B features pillars with a height of 50 μm and a spacing of 220 μm.

## Approach

**Figure 2:**
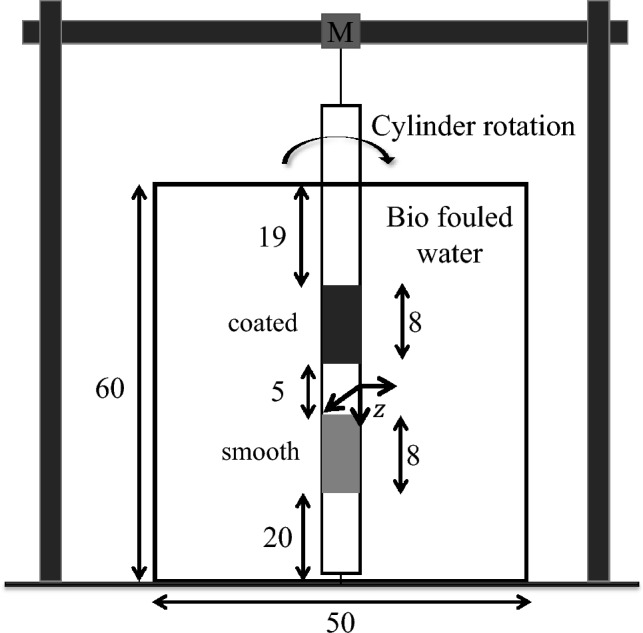
Basic schematic of the experimental setup, which consists of an acrylic tank with dimensions of 50 cm (L) x 50 cm (W) x 60 cm (H) filled with water containing biofouling. A cylinder with a diameter of 6 cm is mounted on the motor and attached to the bottom of the tank via a shaft. Two smooth and bioinspired coated surfaces measuring 10 cm (H) x 19 cm (L) are positioned at 18 cm and 32 cm from the tank bottom. An 18 cm gap is maintained at the top and bottom to avoid end effects. The Stepper motor (M) controls the cylinder rotation, modifying the flow regime.

To investigate the impact of flow regime on the anti-biofouling properties of bioinspired coatings, we employed a canonical flow setup. This setup consisted of a rotating cylinder situated in a quiescent, biofouled aqueous environment contained within an acrylic tank measuring 50 cm (L) $\times$ 50 cm (W) $\times$ 60 cm (H), as shown in Fig. 2. A steel threaded shaft with a diameter of 10 mm was introduced through an 80 cm long PVC pipe with end caps, enabling rotation by a stepper motor at varying RPMs of 180 (turbulent) and 40 (laminar). The shaft was connected to the base using a bearing, and a gap of 1.5 cm existed between the end caps and the bottom of the tank. The stepper motor was supported by 80/20 aluminum beams.

We subjected the bioinspired coated surfaces under two different flow regimes: turbulence and laminar conditions. A rotating cylinder initiated flow in biofouled water at two distinct Reynolds numbers based on the cylinder diameter, denoted as $Re_D = \rho D^2 \omega /2 \mu$, where $\rho$ denotes the density of water, *D* is the diameter of the cylinder, $\omega$ is the angular velocity of the cylinder, and μ represents the dynamic viscosity. The chosen Reynolds numbers, 6.7 × 10$^4$ and 1.5 $\times$ 10$^4$, enable the examination of the efficacy of bioinspired structures in preventing biofouling under both turbulence and laminar flow conditions^23^, respectively. This study focused on bioslime, which constitutes the primary stage of biofouling and consists of primary and secondary colonizers^8^. Bioslime is microscopic and not visible to the naked eye, and it can form within a couple of days of exposure^7^. However, tertiary and macroscopic fouling attach to the surface to feed on the primary and secondary colonizers. Preventing the formation of bioslime would help avoid the large-scale drag forces induced by macroscopic fouling.

### Experimental procedure

Two sets of experiments were conducted to examine the effect of flow regime on biofouling of bioinspired coatings. In the first set, a pattern A bioinspired coated surface and a smooth surface were mounted at specific locations on the rotating cylinder, as illustrated in Fig. 2, and rotated at desired RPM for 7 days in quiescent biofouled water. After the seventh day, the water was drained, and loose biofouling was removed using clean tap water. Then, the surfaces were immersed in 1.25% glutaraldehyde in 0.1 M sodium cacodylate buffer which serves as a fixative for bio-organisms, and imaged using Scanning Electron Microscopy (SEM). In the second set of experiments, a new PVC pipe was utilized, free from fouling and debris. The pattern B bioinspired coated surface and a smooth surface were mounted on the cylinder and operated for 7 days. The same methodology was used for SEM imaging. The laminar flow regime experiments followed the same procedure but with a lower stepper motor RPM. To ensure that the biofouling in the water was consistent among the cases, the water in the acrylic tank was changed at the beginning of each experiment, with a ratio of 60% biofouled water and 40% tap water.

### SEM imaging

The fixed biofouled surfaces were rinsed and dried mechanically to prepare them for observation using the FEI Teneo scanning electron microscope (SEM) at Purdue University’s Department of Agriculture and Biology SEM facility. From the 10 cm x 16 cm surface, eight samples, each measuring 1.5 cm x 1 cm, were collected and rinsed before being subjected to SEM imaging. The SEM images were captured in low-vacuum mode using a 5 kV electron beam with a current of 0.4 nano Amperes and a water pressure of 50 Pa, and 15 images were taken at 350 magnification for each sampled location. To quantify the amount of biofouling present in the images, the SEM images were manually processed using Fiji (ImageJ) software to mask the fouling. Fouling sizes of less than 20 microns are disregarded in the present calculation based on the chosen magnification of SEM imaging.

## Results and discussion

For each set of experiments, we pumped out the biofouled water, inspected the surfaces for fouling every 3, 5, and 7 days, and then pumped in fresh water after each inspection. We observed no visible formation of biofouling at the end of 7 days. As a preliminary test, we used Gram’s stain protocol^24^, employing Gram’s Iodine and crystal violet solution, to test the presence of bioslime on pattern A, B, and smooth surfaces exposed under the turbulence regime. Upon qualitative examination using a Stereo microscope ( Figure S1 in the Supplementary Information), we noted fewer purple patches on pattern A compared to pattern B and smooth, indicating the antifouling behavior of pattern A. However, it should be noted that Gram’s stain protocol has limited reactivity with Gram-positive bacteria, and the color of the stains depends on the illumination of the Stereomicroscope. To quantify and visualize the various types of bioslime on the bioinspired and smooth surfaces, we employed SEM and preserved the bioslime on the surfaces using the methodology described in the section SEM imaging.

### Biofouling formation under turbulent and laminar flow regimes

**Figure 3:**
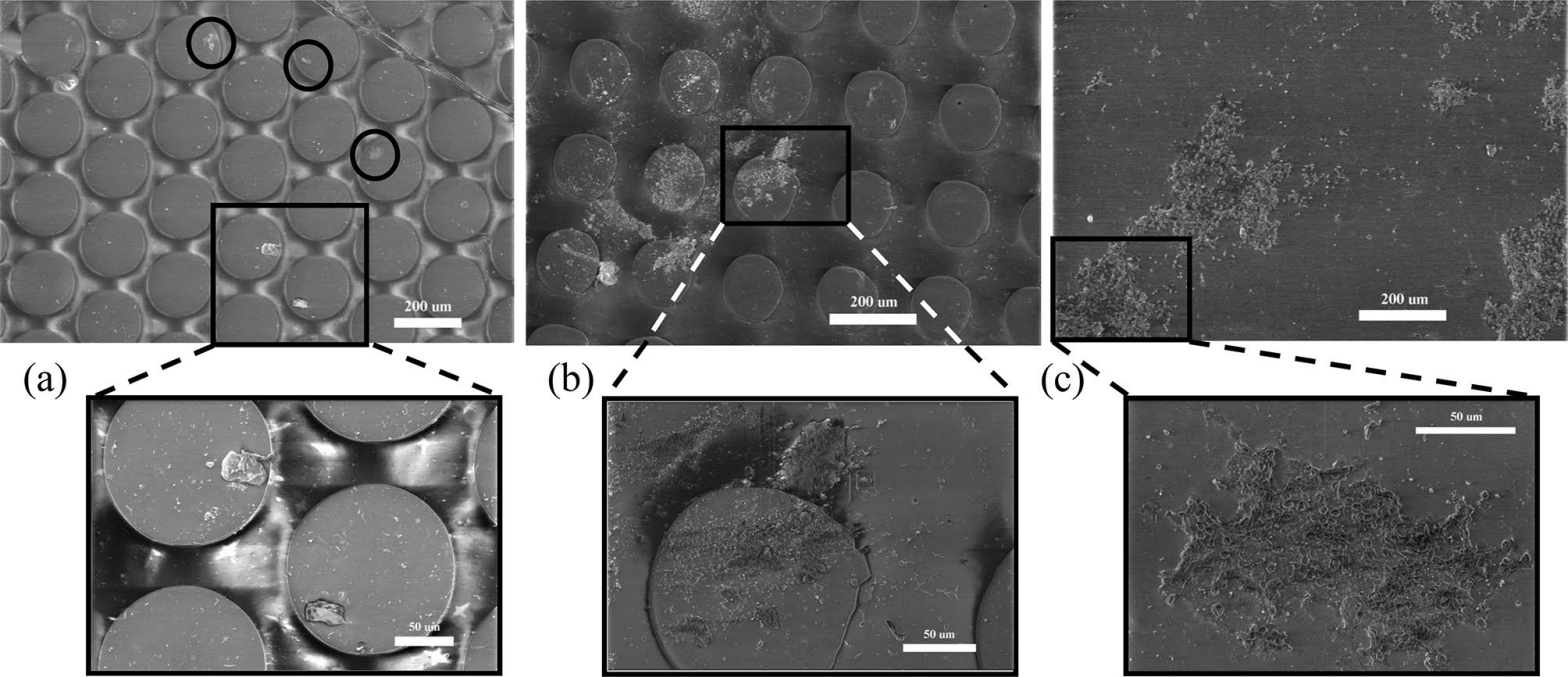
Scanning Electron Microscope (SEM) images showing fouling on the surfaces with varying patterns of microsurfaces under turbulent flow. (**a**) Pattern A coating, (**b**) Pattern B coating, and (**c**) smooth surface; the scale bars in (**a**)–(**c**) are 200 μm, and the zoomed regions are 50 μm.

The Methods section outlines the experimental setup and SEM imaging methodology. Figure 3 illustrates the SEM images of biofouling on coating with patterns A and B and hydrodynamically smooth surfaces without structures under turbulent flow conditions. The zoomed regions provide a detailed view of the features attached to the surfaces. At least 48 randomly selected images for each case were collected from eight locations, covering the entire sample exposed to biofouling-infested water for seven days. These images depict the overall trend of biofouling observed on various surface configurations.

**Figure 4:**
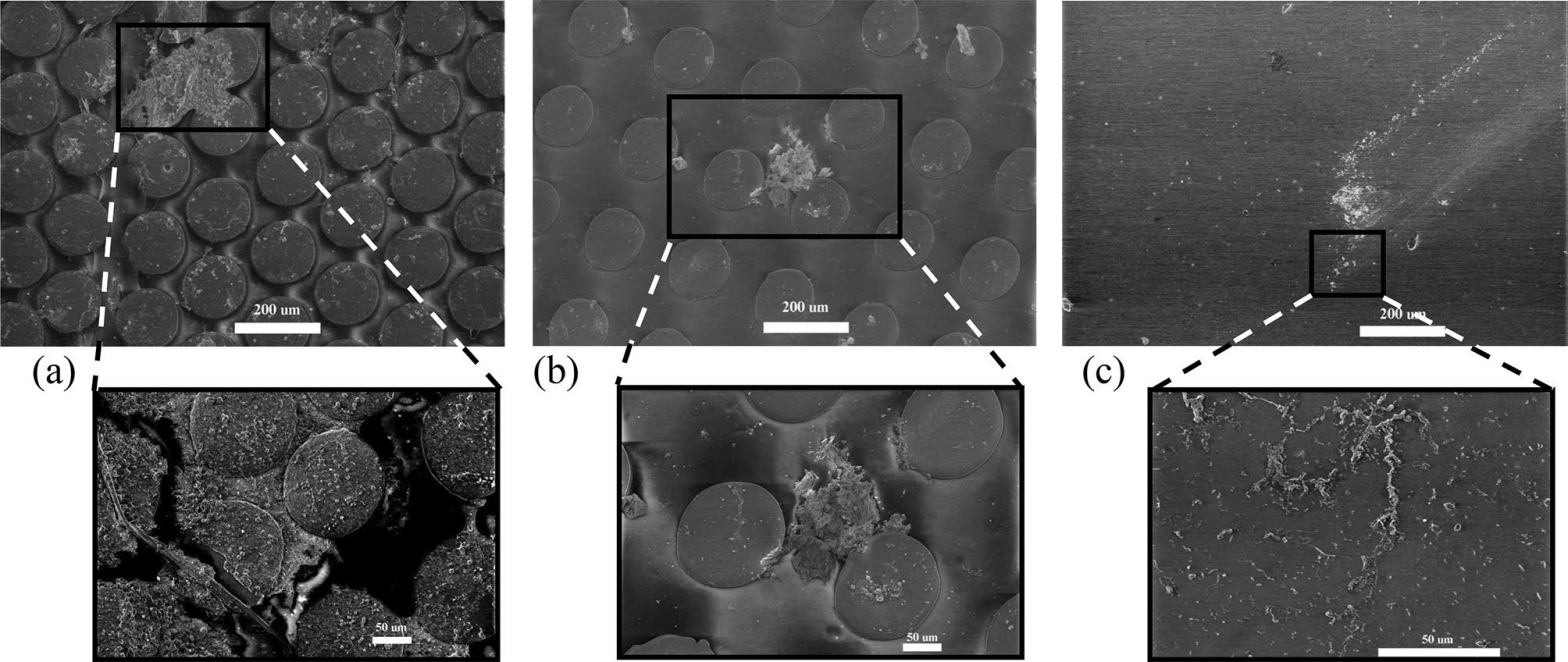
Scanning Electron Microscope (SEM) images showing fouling on the surfaces with varying patterns of microsurfaces under laminar flow regime: (**a**) Pattern A coating, (**b**) Pattern B coating, and (**c**) smooth surface; the scale bars in (**a**)–(**c**) are 200 μm and inset figures are 50 μm.

The coatings (patterns A and B) underwent distinct biofouling characteristics. Pattern A coating had small clusters of diatoms and bacterial colonies, each measuring less than 50 μm. These clusters settled on the pillar surfaces and the spaces between the pillars. Conversely, pattern B configuration had larger patches of biofouling with a size greater than or equal to 80 μm. The zoom in Fig. 3b highlights the significance of the spacing between pillars and pillar height in determining the formation of biofouling clusters that span both the pillar surface and the spaces between them.

Observations of biofouling on a smooth surface indicate the formation of larger clusters consisting of bacterial and algae colonies, as depicted in Fig. 3c, with a size ≥200 μm. According to Martin-Rodriguez et al.^8^, the presence of such microbial and fungal colonies attracts secondary and tertiary predatory species and macro biofouling, which can significantly affect the drag and lead to regular maintenance of marine vehicles. Therefore, it is crucial to appropriately consider the spacing and height of the bioinspired structures to minimize biofouling and its detrimental effects on the structure.

**Figure 5:**
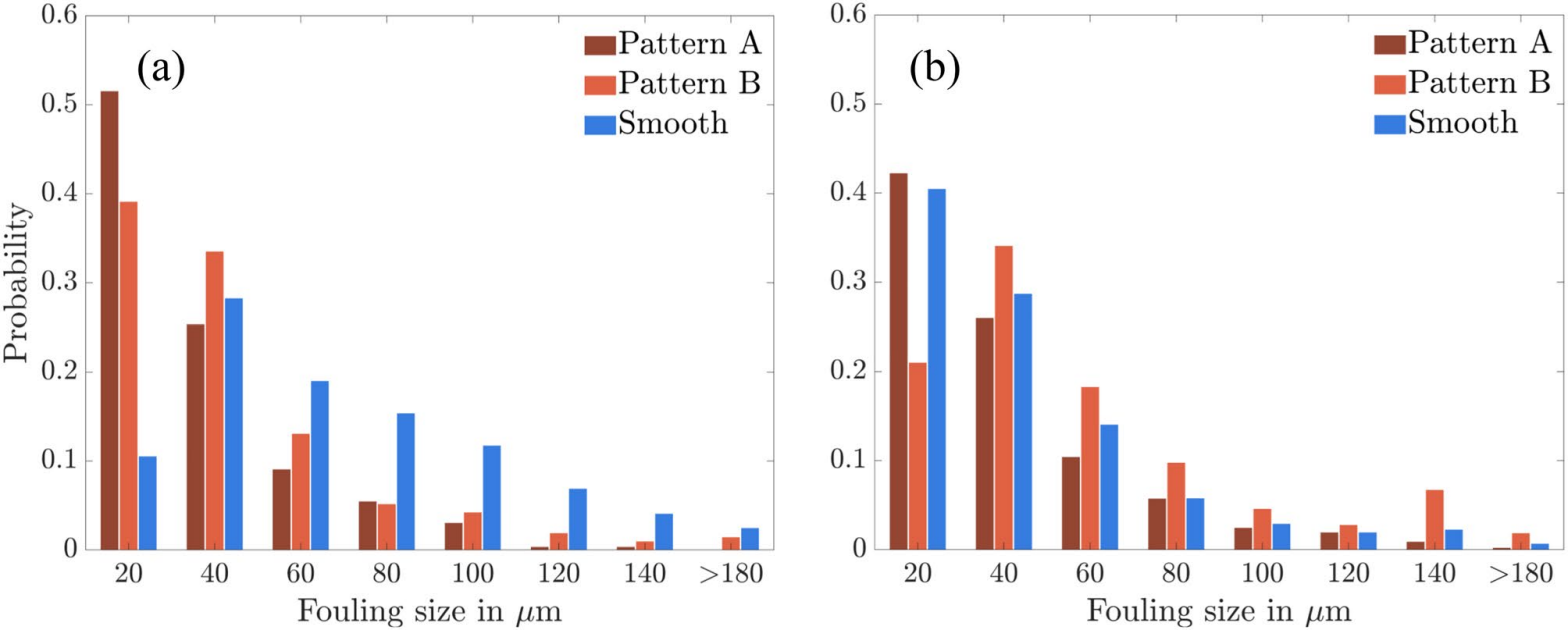
Probability distribution biofouling of size, $s_i\ge 20$ μm. (**a**) turbulent and (**b**) laminar flow conditions for the pattern A and B coating and smooth surface obtained over eight sampled locations and at least 48 SEM images.

The deposition pattern of biofouling on the three surface configurations (coating with patterns A and B and smooth case) is distinct under laminar flow compared to turbulence conditions. The SEM images in Fig. 4 demonstrate that biofouling deposition on these surfaces ranges from small sizes ($\approx 40$ μm) to large patches ($\ge 200$ μm), which is more clearly observable under laminar flow conditions. In the coating with pattern A, fouling of the order of $\approx 200$ μm is formed between the pillars, covering the space between them. Additionally, biofouling is present around the irregularities present on the surface due to the manufacturing process under laminar flow conditions but not under turbulence conditions.

In pattern B coating, biofouling appears to form similar features in turbulent and laminar flow conditions, covering the pillar surface and the space between them. However, in the smooth case (Fig. 4c), biofouling seems to follow a flow pattern and forms large clusters of bacterial colonies formed by small subgroups (Fig. 4c zoom). The dissimilarity in biofouling between laminar and turbulent flow conditions could be attributed to various factors, including Reynolds stress distribution in the two flow regimes at the interface between the bioinspired features, the inner and outer exchange and the directional flow fluctuations under turbulence affecting the adhesion and settlement of biofouling on the surface.

### Quantification of antifouling effects of bioinspired surface coating

The likelihood of detecting biofouling with a size of $s_i$ can be determined as follows

1 $$\begin{aligned} p({s_i}) = \frac{\sum _{t=1}^{t=N}\sum _{s_i-10}^{s_i+10}n(s_i)_t}{\sum _{t=1}^{t=N}\sum _{s_i=20\mu m}^{s_i\le 200\mu m}n(s_i)_t} \end{aligned}$$

where $n(s_i)_t$ indicates the number of occurrences of biofouling size, $s_i$, in the $t_{th}$ image, and *N* is the total number of SEM images collected at 350X magnification. The numerator of the equation adds up the instances of biofouling sizes within a $\pm 10$ μm range around $s_i$ across all images. In contrast, the denominator sums up the occurrences of biofouling sizes ranging between 20 μm and 200 μm across all images. Essentially, the equation determines the proportionate frequency of biofouling of a particular size in the given size range. By analyzing the resulting probability distribution, valuable insights can be obtained into the probability of biofouling of various sizes appearing on the surfaces in question.

The probability distribution of biofouling formation for different surface configurations is illustrated in Fig.5. The 350X magnification helps to concentrate on biofouling visualization in the range of 10-20 μm. This range was chosen to investigate the development of smaller organisms into larger ones, which attract tertiary and macro fouling as prey. Observations reveal that the coating with pattern A has a higher probability of fouling size of 20 μm, with frequent occurrences of approximately 0.5 and 0.4 for turbulence (Fig.5a) and laminar (Fig. 5b) flow regimes. Nonetheless, the likelihood of discovering larger fouling sizes declines, and the possibility of finding fouling sizes $\ge 100$ μm is negligible for both turbulence and laminar regimes. Although the laminar flow conditions have a higher probability than the turbulence regime, the number of occurrences of higher fouling size is minor.

Patterns A and B coatings do not show significant differences in the likelihood of finding fouling sizes less than 40 μm. Notably, Pattern A’s occurrence frequency is lower in turbulence than laminar as biofouling size increases. However, it shows a higher value than pattern A. Additionally, pattern B exhibits a significant probability at biofouling sizes $\ge 100$ μm. The probability distribution of biofouling sizes on different surfaces suggests that specific configurations are more prone to biofouling of particular sizes, indicating that the physical characteristics of the surface can influence the adhesion and settlement of biofouling organisms. However, a crucial factor is a variation in probability between the two flow regimes for the same configuration. Unlike the pattern A coating, the likelihood of biofouling in the turbulence flow regime for pattern B with size $\ge 100$ μm is similar to the values in the laminar flow regime. This emphasizes the link between microsurface pattern and flow regime in designing bioinspired coatings to mitigate biofouling (Fig. 5).

For a smooth surface, the probability of encountering biofouling with sizes smaller than or equal to 40 μm is significantly lower than that of surface coating configurations. However, the likelihood increases for biofouling sizes greater than 40 μm, indicating that if biofouling occurs, it can grow rapidly to larger sizes within a few days of exposure with no external influence. Also, Fig. 5a,b reveal that the probability distribution for the smooth surface displays a comparable trend and has similar values for both turbulence and laminar flow conditions. This implies that flow regimes have a lower impact on biofouling on a smooth surface than on bioinspired coatings (Fig.5).

**Figure 6:**
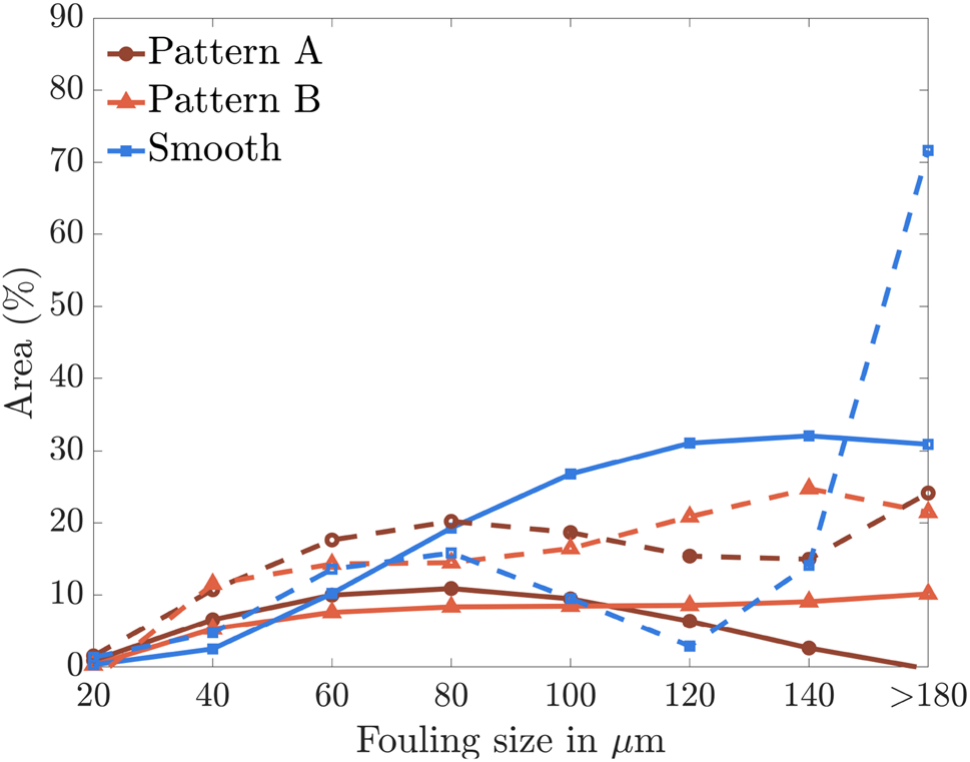
Percentage area of biofouling over different biofouling sizes for the pattern A () and pattern B () coatings and smooth () surfaces obtained over eight sampled locations and at least 60 images; Solid and dashed lines indicate turbulent and laminar flow regimes.

Although the probability of having biofouling with sizes $\le 40$ μm is greater for the pattern A and B, the percentage of area covered by biofouling of this size is less than 10%, as illustrated in Fig. 6. Nonetheless, this is still a substantially lower percentage of biofouling area than that observed on the smooth surface at 80 μm. Pattern A coating displays a decrease of approximately 45% in biofouling area percentage compared to the smooth surface at 80 μm. It should be noted that under the laminar flow regime, the area covered by biofouling is higher in the pattern A than in the turbulence regime. Interestingly, under the laminar regime, pattern A coating shows around 25% biofouled area for fouling sizes greater than 180 μm due to the coalescence of debris with the biofouling. Also, under the turbulence flow regime, the pattern B coating shows a consistent increase in biofouling area with increasing size and remains nearly constant after 80 μm biofouling size. Under the laminar flow regime, the percentage of biofouling area increases and shows similar percentage as of pattern A in laminar flow regime (Fig. 6).

**Figure 7:**
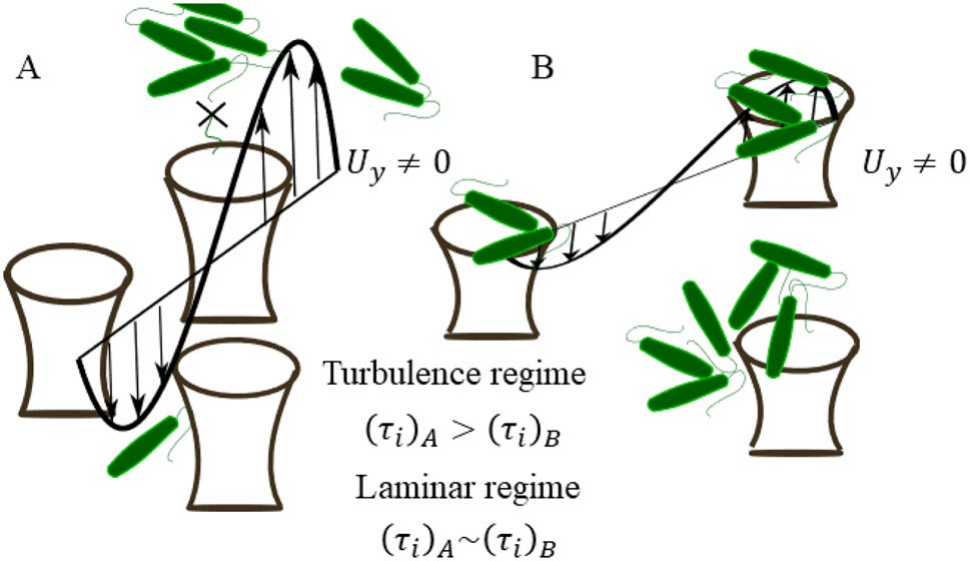
Schematic of the anti-biofouling properties of pattern A and B bioinspired coating under different flow regimes.

The solid blue line in Fig. 6 represents the surface subjected to a turbulence flow regime and exhibits a significantly higher percentage of biofouling than other cases, including the smooth surface under the laminar flow regime. This observation reinforces the importance of flow conditions on the onset of biofouling.

It is worth noting that there is roughly a Stokes flow within the micropillars. At the interface of the micropillars and the boundary layer, there is an interplay between the highly viscous-dominated flow and the inertia-dominated boundary layer. Bocanegra et al.^1^ showed that micropillar-based coatings promote unsteady blowing and suction, resulting in higher wall-normal velocity fluctuations. However, the characteristics of the micropillars array and size are linked to the stresses that may help to reduce biofouling. Here, we derive a basic relationship between the wall-normal fluctuations and the ratio of the spacing and height of the micro-pillars; details are given in the Methods section.

Dierich et al.^25^ observed that in the inner layer of the wall, the velocity profile is weakly influenced by the curvature of the cylinder and can be approximated as flow over a flat plate. Hence, the shear stress, $\tau _{xy}$, within the boundary layer is given by a laminar ($\tau _{lam}$) and turbulent ($\tau _{turb}$) contributions, given as follows:

2 $$\begin{aligned} \tau = \tau _{lam} - \tau _{turb} = \mu \partial U_{x}/\partial y - \rho \overline{u'_{x}u'_y}, \end{aligned}$$

where $U_{x}$ is the averaged velocity in the tangential direction to the cylinder surface, $u'_{y}$, and $u'_x$ are the instantaneous velocity fluctuations, and μ is the dynamic viscosity of the fluid. The near-wall contribution of turbulent shear stress is absent for the smooth surface, leading to $\tau = \mu \partial U_{x}/\partial y |_{y=0}$. See additional details in Methods section.

In contrast to the smooth surface, the coatings had lower biofouling percentages under the turbulence regime. Within bioinspired structures, the Reynolds number $Re_{in}=\rho U_{x,in} s/\mu$, based on the spacing, *s*, is very low, on the order of 1, i.e., dominant viscous effects. This makes the Stokes flow equations appropriate for describing flow within the bioinspired structures. However, the flow regime outside these structures may vary depending on the outer length scales and flow velocity. At the interface of the bioinspired structures, the boundary layer equations satisfy the Laplacian pressure equation. This approach allows determining the shear stress, $\tau _i$, at the interface between the bioinspired structures and the boundary layer.

3 $$\begin{aligned} \tau _i = \tau _w + \frac{s}{h} \rho \overline{u^{'2}_y} \end{aligned}$$

where $\rho$ is the fluid density, $\tau _w$ is wall shear stress, and $\overline{u^{'2}_y}$ is the wall-normal velocity fluctuations at the interface of the bioinspired structures and boundary layer. Bocanegra et al.^1^ found that bioinspired structures induce wall-normal fluctuations at their interface, which affect the shear stress. The relation between the shear stress and the $\frac{s}{h}$ ratio of the bioinspired structures is demonstrated by Eq. 3, and Fig. 6 reveals that the pattern B configuration, with a $\frac{s}{h}$ ratio of 4.4, experiences higher biofouling than pattern A coating with a ratio of 2.1. The formulation highlights that both the $\frac{s}{h}$ ratio and the wall-normal fluctuations impact the wall shear stress, but further inspection using, e.g., direct numerical simulations is needed to determine the precise relationship between them and to analyze the flow within the bioinspired structures. An inverse relationship between the $\frac{s}{h}$ ratio and wall-normal fluctuations may exist since increasing the ratio results in a higher Reynolds number ($Re_{in}$) within the bioinspired structures, departing from the Stokes flow assumption. The wall-normal velocity fluctuations become zero in the laminar regime, equaling $\tau _i$ and $\tau _w$. Consequently, the value of $\tau _i$ becomes independent of the $\frac{s}{h}$ ratio. This is indicated by the comparable percentages of biofouling area observed for the pattern A and B coatings, as illustrated in Fig. 6. Finally, a schematic of patterns A and B depicting basic anti-biofouling properties is illustrated in Fig. 7.

## Discussion

Our study is the first experimental investigation to explore how flow regimes affect the effectiveness of bioinspired structures as anti-biofouling coatings. Our findings underscore the crucial role of flow regime, spacing, and patterns in designing bioinspired coatings for optimal biofouling prevention. In particular, SEM images from the pattern A coating demonstrate that biofouling with sizes of $\le 80$ μm is predominant in the laminar and turbulent flow regimes.

The experimental results reveal that the percentage of biofouling area was higher under laminar flow compared to turbulent conditions. Specifically, for a biofouling size of 80 μm, the pattern A coating exhibited about 25% biofouling under laminar flow and approximately 15% under turbulent conditions, highlighting the critical role of flow conditions in biofouling. The pattern B coating showed a reduced likelihood of forming small biofouling sizes compared to the pattern A, indicating the significance of bioinspired structures’ spacing and height. However, pattern B coating exhibited a higher likelihood of forming larger biofouling sizes ($\ge 80$ μm), with larger patches ($\ge 180$ μm) responsible for 40% of the biofouled area. Unlike the pattern A, the pattern B did not show significant variation in the biofouled area between laminar and turbulent regimes, which can be attributed to the reduced normal Reynolds stresses in the wall-normal direction with an increasing *s*/*h* ratio. The smooth surface configuration shows a higher probability of forming large biofouling sizes ($\ge$80 μm); biofouling merged with debris to form more extensive biofouling patches, facilitating a larger surface area for other biofouling to form clusters.

The findings of our study highlight the crucial role of flow conditions in managing biofouling, as well as the interplay between the spacing and height of bioinspired coatings and the flow regimes. The results evidence the need to comprehensively consider these factors when designing anti-biofouling coatings.

## Theoretical arguments

The wall-normal Navier-Stokes equations in the boundary layer region in the vicinity of the surface, which encompasses the flow above the bioinspired structures, are simplified as follows:

4 $$\begin{aligned} \frac{1}{\rho }\frac{\partial P}{\partial y} = - \frac{\partial \overline{u{'}_y^{2}}}{\partial y} \end{aligned}$$

The left-hand side indicates the vertical pressure gradient, while the right-hand side represents the wall-normal gradient of the Reynolds stress, $\overline{u{'}_y^2}$, where the overline denotes the ensemble average. Similarly, in the streamwise direction, the simplified equation can be expressed as follows:

5 $$\begin{aligned} \frac{1}{\rho } \frac{\partial P}{\partial x} = \nu \frac{\partial ^2 U_x}{\partial y^2} - \frac{\partial \overline{u'{_x} u{'}_y}}{\partial y} \end{aligned}$$

The left-hand side term of Eq. 5 represents the pressure gradient in the streamwise direction, while the right-hand side term is a combination of the viscous stress, $\nu \frac{\partial ^2 U_x}{\partial y^2}$, and the Reynolds shear stress, also known as turbulence shear stress, -$\frac{\partial \overline{u'_x u'_y}}{\partial y}$. The Eq. 5 can be written as

6 $$\begin{aligned} \frac{1}{\rho } \frac{\partial P}{\partial x} = \frac{\partial \tau }{\partial y} \end{aligned}$$

where $\tau = \tau _w +\tau _{turb}$. Within the bioinspired structures, there is a Stokes-like flow. Hence, the simplified governing equations are as follows,

7 $$\begin{aligned} \frac{\partial P}{\partial x} = \mu \left[ \frac{\partial ^2 U_x}{\partial x^2} +\frac{\partial ^2 U_x}{\partial y^2} \right] ; \frac{\partial P}{\partial y} = \mu \left[ \frac{\partial ^2 U_y}{\partial x^2} +\frac{\partial ^2 U_y}{\partial y^2} \right] \end{aligned}$$

The $U_x$ and $U_y$ are the time-averaged mean velocities in streamwise and wall-normal directions. Due to the assumption of Stokes flow, the Laplacian of pressure within the bioinspired structures is zero, i.e., $\nabla ^2 P = 0$. The boundary conditions must be satisfied at the interface of the bioinspired structures and the boundary layer, leading to the deduction of Eq. 4 and 5 as follows:

8 $$\begin{aligned} \frac{\partial ^2 \tau }{\partial x \partial y} - \rho \frac{\partial ^2 \overline{u{'}_y^2}}{\partial y^2} = 0 \end{aligned}$$

After taking the partial derivative with respect to *x* and *y*, the summation must equal zero. Integrating this over the spacing between the bioinspired coatings, *s*, we arrive at Eq. 8.

9 $$\begin{aligned} \frac{\partial \tau }{\partial y}|_{y=h} = \rho \frac{\partial ^2 \overline{u_{y'}^2}}{\partial y^2} \times s \end{aligned}$$

Additionally, the gradients within the bioinspired structures can be approximated by expressing the shear stress at the interface and wall as $\frac{\partial \tau }{\partial y} = \frac{\tau _{i}-\tau _{w}}{h}$ and the wall-normal gradient of the Reynolds stress as $\frac{\partial ^2 \overline{u{'}_y^2}}{\partial y^2} = \frac{\overline{u{'}_y^2}-0}{h^2}$, respectively. Here, $\tau _i$ and $\tau _w$ represent the shear stress at the interface and the wall, while wall-normal fluctuations at the wall are zero.

Upon further simplification, Eq. 9 can be expressed as follows:

10 $$\begin{aligned} \tau _i = \tau _w + \frac{s}{h} \rho \overline{u{'}_y^2}. \end{aligned}$$

## Supplementary Information

Supplementary Information.

## Data Availability

The datasets generated during and/or analysed during the current study are available from the corresponding author on reasonable request.
