# Supplementary material for: Impact of flow regime on the performance of anti-biofouling coatings
Source: Sci Rep. 2023 Jun 12;13:9501. doi: 10.1038/s41598-023-36736-7 (PMC10260965; doi:10.1038/s41598-023-36736-7)
Supplement: Supplementary file 1 — Supplementary Information. [file 41598_2023_36736_MOESM1_ESM.pdf]

Impact of flow regime on the performance of anti-biofouling coatings

Venkatesh Pulletikurthi[1,*], Helber Antonio Esquivel-Puentes[2], Shyuan Cheng[3], Leonardo P. Chamorro[3], and Luciano Castillo[1]

[1]School of Mechanical Engineering, Purdue University, West Lafayette, IN 47906
[2]Department of Agricultural and Biological Engineering, Purdue University, West Lafayette, IN 47906
[3]Department of Mechanical Science and Engineering, University of Illinois Urbana-Champaign, IL 61801
[*]vpulleti@purdue.edu

Supplementary Information

Supplementary Figure 1

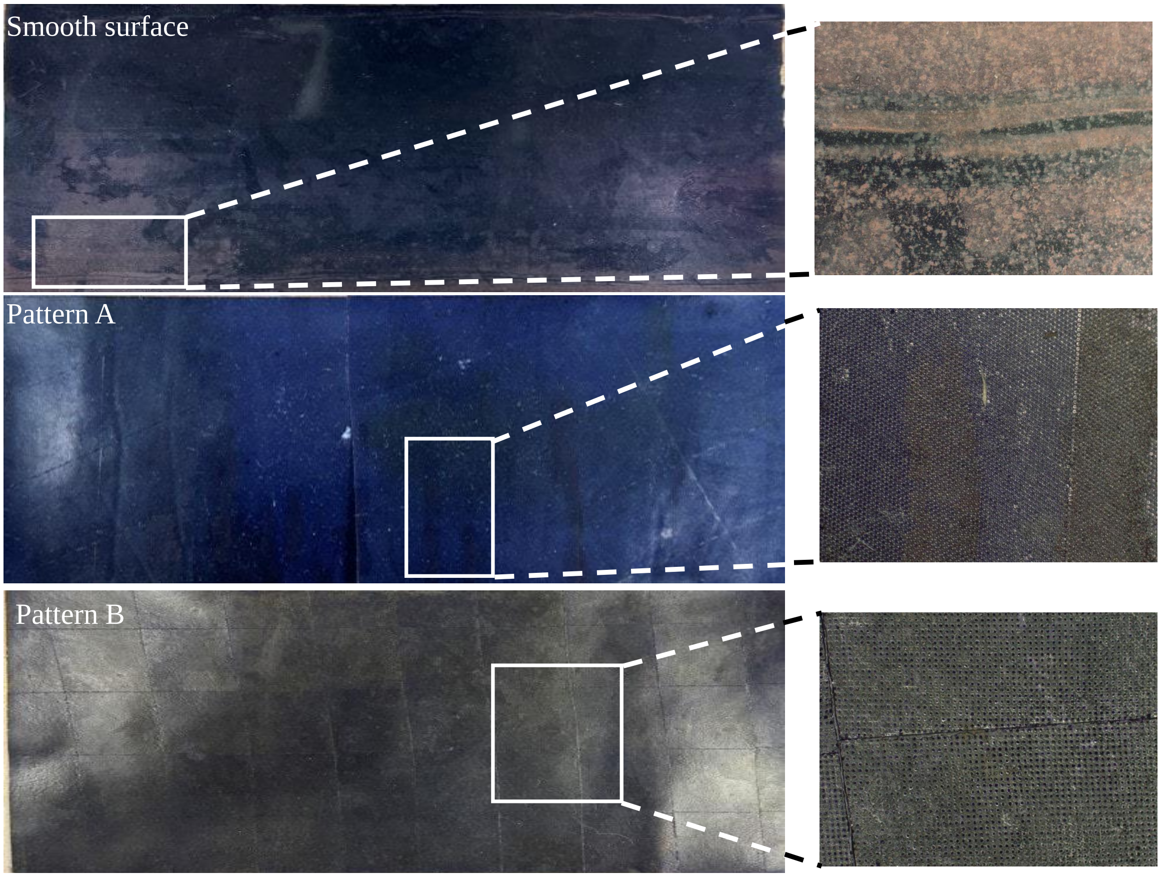

Figure S1. Biofouled surfaces under turbulence regime after performing Gram's staining protocol and the inset images are observed under Stereo microscope

Figure S1 displays the biofouled surfaces after undergoing Gram's chemical staining procedure. The staining procedure involves the penetration of crystal violet and iodine into the protein membrane of Gram-positive organisms, forming a purple/blue complex known as Crystal Violet Iodine (CVI). As illustrated in figure S1, there are purple/bluish patches on patterns A, B, and smooth surfaces. The boundaries of the bioslime were distinguished using a Stereo microscope and are shown in the insets.
